# Pilonidal sinus of breast, a case report with literature review

**DOI:** 10.1016/j.amsu.2021.103138

**Published:** 2021-12-02

**Authors:** Abdulwahid M. Salih, Zuhair D. Hammood, Hiwa O. Abdullah, Fahmi H. Kakamad, Bakhan Sharif Ali, Karzan M. Salih, Berwn A. Abdulla

**Affiliations:** a Smart Health Tower, Madam Mitterrand Street, Sulaimani, Kurdistan, Iraq; b College of Medicine, University of Sulaymaniyah, Sulaymaniyah, Iraq; c Kscien Organization, Hamdi Str, Azadi Mall, Sulaimani, Kurdistan, Iraq; d Iraqi Board for Medical Specialities, Sulaimani Center, Sulaymaniyah, Iraq

**Keywords:** PNS, Breast, Acquired infection

## Abstract

**Introduction:**

Pilonidal sinus (PNS) is an acquired inflammatory infection that mostly occurs in the sacrococcygeal region. This study aims to present a case of breast pilonidal sinus with a brief literature review.

**Case presentation:**

A 31-year-old female presented with two painful right breast lumps for about one-month duration. Ultrasound (US) examination revealed heterogeneous areas with minimal inflammation at the right breast. There was also a nipple inversion, retro areolar duct dilatation, skin fistula, and reactive inflammatory level I axillary lymph nodes, suggesting inflammation/infection. Wide local excision was done. Histopathological examination revealed duct ectasia with a focus of sclerosing adenosis and a single hair shaft located in the breast tissue.

**Discussion:**

Authors encourage surgical treatment (excision and primary closure) due to high recurrence risk after aspiration, medical treatment, and slow response of the abscess to antibiotic administration. Gender, family history, smoking, overweight, sinus size, poor hygiene, and surgical technique are among the risk factors that play a role in recurrence.

**Conclusion:**

Breast PNS is a very rare and atypical variant of PNS that may occur due to nipple inversion, hormonal effect, poor hygiene, tight brassieres. Surgical treatment is the best option to reduce recurrence risk.

## Highlights

• Pilonidal sinus disease (PSD) is an acquired inflammatory infection.
• The main clinical manifestations include abscess formation or chronic persistent secretion.
• The region of the disease is sacrococcygeal area.
• In this paper, a case of PSD has been reported and discussed.

## 1 Introduction

Pilonidal sinus (PNS) is an acquired inflammatory infection that occurs due to the penetration of hair shafts into the epidermis of the skin [1]. Accumulation of foreign bodies and hair fragments in moist body parts are considered the primary cause of the infection, which is characterized by tract formation, pus collection, tenderness, and local pain. It is mostly asymptomatic, and the appearance of a small-sized ditch on the skin surface can be the only sign of its occurrence. The main clinical manifestations include abscess formation or chronic persistent secretion [2]. PNS mainly affects the intergluteal cleft or sacrococcygeal regions but rarely can occur in the other parts of the body [3]. The incidence of PNS is about 0.07% and comprises %15 of perianal diseases. Commonly susceptible individuals to PNS are those aged between 10 and 40 years with a men predominance of 3–4 times [4]. Sedentary lifestyle, excessive hair, poor hygiene, deep natal cleft, direct contact with broken hair due to occupations like hairdressing and animal grooming are major risk factors of PNS [1].

The aim of this paper is to report a case of PNS occurring in the breast of a young lady. The paper has been written in line with SCARE 2020 guidelines [5].

**Patient information:** A 31-year-old female patient presented with two painful right breast lumps for about one-month. She had received antibiotics and underwent aspiration, but afterward, she developed continuous yellowish thick discharge from the aspiration site. She is G2P2A0 and has lactated for two years. She doesn't have any chronic disease; she has undergone two cesarean sections and a tympanoplasty.

**Clinical Examination:** There was a chronic discharging sinus at 9–10 o'clock of the right breast, surrounded by indurated erythematous skin, with a serosanguinous foul odored discharge, and associated with a retracted nipple.

### 1.1 Diagnostic assessment

Ultrasound (US) examination showed heterogeneous areas with minimal inflammation at the right breast; a long collection sized (30*5mm) extending from the retro areolar region towards 9 o'clock, a small collection sized (13*7mm) located towards 10 o'clock, and another collection (15*6mm) towards 12 o'clock. There was also nipple inversion, retro areolar duct dilatation, a skin fistula at 8 o'clock, and reactive inflammatory level I axillary lymph nodes, suggesting inflammation/infection (U2). Left breast examination was normal, apart from a small 10mm simple cyst at 2 o'clock.

### 1.2 Therapeutic intervention

Because the top differential diagnosis was chronic inflammation (granulomatous mastitis), the patient was prescribed prednisolone tab 10mg twice per day and methotrexate 2.5mg once per day for two months. Unfortunately, there was no significant improvement, and hence surgery was decided. Surgical intervention was done in the form of wide local excision, and the tissue was sent for histopathological examination. The histopathology revealed duct ectasia with a focus of sclerosing adenosis and a single hair shaft in the breast tissue.

**Follow up:** The post-operative period was uneventful, and the patient continued taking prednisolone 20mg once per day.

## 2 Discussion

PNS is an inflammatory disease that commonly affects the sacrococcygeal or perianal area due to the invasion of hair particles into the adjacent skin [1,6]. It is more frequent in males than females and often present at the age of 10–40 years [4]. The four major reasons for the formation of PNS are hair penetration to the skin, skin folding in the natal cleft, hormonal effect, and continuous pressure on atypical areas [7]. Abscess and chronic persistent secretion are regarded as the major clinical manifestations of the disease [2]. The current case was a 31-year-old female with two painful lumps at her right breast with a continuous serosanguinous foul odored discharge.

In the past, PNS was thought to be a congenital disease, until an acquired hypothesis was suggested by Patey and Scarff. The hypothesis stated that the disease is acquired through the gradual penetration of hair into the subcutaneous tissue of the adjacent skin and developing into a long-term low-grade infection [7,8]. The jobs most commonly associated with sacrococcygeal PNS were farming followed by irregular occupations and salespeople. Other occupations, like barbers, through their dealings with hair, were at risk for another type of PNS, like the interdigital PNS [8]. According to Harlack et al., being hairy and sitting down for more than 6 hours daily increases the chance of PNS infection [9]. While another study by Hama Shareef et al. revealed that excessive body hair is not always needed to develop PNS and they depended on the fact that the affected area can be hairless such as the intermammary region of females [10]. The current case is in accordance with Shareef et al. because the PNS was originated from the right breast, which is a hairless region.

Some other studies believed that nipple inversion which previously found to be benign is responsible for PNS formation by local hair follicles bending or hair trapping [1,10]. There is correlation between the present study with the other two previous studies because there is also nipple inversion, retro areolar duct dilatation and skin fistula in the current case.

Some relevant ways that have been proposed to prevent breast PNS include weight loss and hair penetration prevention by wearing tightly woven clothing and fitting brassieres [1].

Regarding the diagnosis of perianal PNS, clinical examination is usually sufficient, but diagnosis of atypical PNS is more difficult. Chronic discharging sinus is reliable proof for the physician to diagnose all atypical PNS cases except umbilical and interdigital PNS [9]. The current case had a chronic discharging sinus with a serosanguinous foul odored discharge at the right breast. Ultrasound examination showed minimal inflammation at some regions of the right breast. In addition, nipple inversion, retro areolar duct dilatation, and skin fistula were also seen.

There are a lot of surgical and non-invasive modalities in the management of PNS including incision and drainage, lying open, marsupialization, excision and primary closure, different injections, and laser ablation. Furthermore, non-operative techniques such as injection of healing solutions are another management option. A typical PNS is usually managed by local excision, but post-operative follow-up takes time and is difficult [11]. A preferable surgical therapy includes excision with primary closure, which is more reliable than the previous one due to less time consumption, positive cure rate, less costs, and more patient satisfaction [12]. Non-operative therapies like phenol injection, laser therapy, and ointment sometimes can be used but some studies reported recurrence, failure, and complication development after phenol injection [[13], [14], [15]]. Some studies encourage treatment with surgical therapy (excision and primary closure) due to recurrence after aspiration and medical treatment or slow response of the abscess to antibiotics [1,10]. Gender, family history, smoking, overweight, sinus size, poor hygiene, and surgical technique are among the risk factors involved in recurrence. It is crucial to precisely diagnose PNS and differentiate it from skin-derived infectious diseases, teratoma, and sebaceous gland cyst. If there is no proper follow-up, recurrence and long-term inflammation can increase the risk of carcinogenesis, such as squamous cell carcinoma [16,17]. In the current study, there was no significant improvement following two months of treatment with prednisolone and methotrexate, and hence surgical excision (wide local excision) was decided. The post-operative period was uneventful, and the patient continued taking prednisolone 20mg once per day.

## 3 Conclusion

Breast PNS is a very rare and atypical kind of PNS that may occur due to nipple inversion, hormonal effect, poor hygiene, unfitting or tight brassieres. Surgical treatment is the best option to reduce recurrence risk.

## Provenance and peer review

Not commissioned, externally peer review.

## Conflicts of interest

There is no conflict to be declared.

## Sources of funding

No source to be stated.

## Ethical approval

The manuscript approved by ethical committee of the University of Sulaimani.

## Consent

Consent has been taken from the patients and the family of the patients.

## Author contribution

Abdulwahid M. Salh: major contribution of the idea, literature review, final approval of the manuscript. Zuhair D. Hammood: Surgeon performing the operation, final approval of the manuscript. Fahmi H. Kakamad, Hiwa O. Abdullah: literature review, writing the manuscript, final approval of the manuscript. Bakhan Sharif Ali, Karzan M. Salih, Berwn A. Abdulla: literature review, final approval of the manuscript.

## Registration of research studies

1 Chinese Clinical Trial Registry
2 ChiCTR2100047387
3 Chinese Clinical Trial Register (ChiCTR) - The world health organization international clinical trials registered organization registered platform

## Guarantor

Fahmi Hussein Kakamad is Guarantor of this submission.
